# An extragenital cell population contributes to urethra closure during mouse penis development

**DOI:** 10.1126/sciadv.adp0673

**Published:** 2024-12-06

**Authors:** Ciro Maurizio Amato, Xin Xu, Humphrey Hung-Chang Yao

**Affiliations:** ^1^ Reproductive Developmental Biology Group, National Institute of Environmental Health Sciences, Research Triangle Park, NC 27709, USA.; ^2^ Department of Surgery, Division of Urology, University of Missouri, Columbia, MO 65211, USA.; ^3^ Epigenetics and Stem Cell Biology Laboratory, National Institute of Environmental Health Sciences, Research Triangle Park, NC 27709, USA.

## Abstract

The penis, the organ that bears reproductive and psychological importance, is susceptible to birth defects such as hypospadias or incomplete closure of urethra along the penis shaft. We discover that proper urethral closure in mouse embryos requires a unique mesenchymal cell population originated from outside of the penis. These “extragenital” cells, marked by a lineage marker *Nr5a1*, migrate from the inguinal region into the embryonic penis and facilitate urethra closure by interacting with adjacent periurethral cells via the epidermal growth factor pathway. Ablation of *Nr5a1*^+^ cells leads to severe hypospadias and alters cell differentiation in the penis. This discovery highlights the indispensable role of *Nr5a1*^+^ extragenital cells in urethra closure, shedding light on the biology of penis formation and potential implications for human hypospadias.

## Teaser

An extragenital cell population facilitates penis development and proper urethra closure in mouse embryos.

## INTRODUCTION

External genitalia are one of the few organs that originate as a bipotential primordium identical in embryos of both sexes and eventually differentiate into unique sex-specific organs. During sex differentiation, the external genital primordium transforms into a functionally and anatomically distinct structure: penis in the male and clitoris in the female. This developmental plasticity subjects the external genitalia to congenital abnormalities. Malformation of the penis is among the most common birth defects in the world (*1*). One such defect is hypospadias, where the urethra fails to close at the tip of the penis and, instead, exits somewhere along the shaft. Although hypospadias is common (1:125 male newborns), its etiologies are largely unknown. Gene mutations in androgen receptor (*AR*), 5-α reductase (*SRD5A2*), and steroidogenic factor 1 (*NR5A1*) are linked to hypospadias formation (*2*). Although these predisposing mutations provide a few possible causes for hypospadias, most human hypospadias cases remain unexplained.

Proper penis formation and urethral closure are facilitated by cross-talks between mesenchymal cell populations and the urethral epithelium, situated in the center of the penis. The urethral epithelium is flanked by the periurethra mesenchyme, which secrete various factors that act on the urethral epithelium (*3*–*5*). Neighboring the periurethral cells is an additional group of not well-defined mesenchymal cells that compose the prepuce of the penis (*6*). It is not clear what role these mesenchyme cells in the prepuce play in penis morphogenesis and urethral closure. The fact that 70% of the human cases of hypospadias do not have a known cause highlights the need of developing models to understand the basic biology of penile development. In this study, we applied mouse genetic models, single-cell sequencing, and ex vivo culture systems to discover cell types of the penis and the interactions among these cells in facilitating proper urethral closure.

## RESULTS

### Identification of an extragenital cell population in the penis

**Fig. 1.**
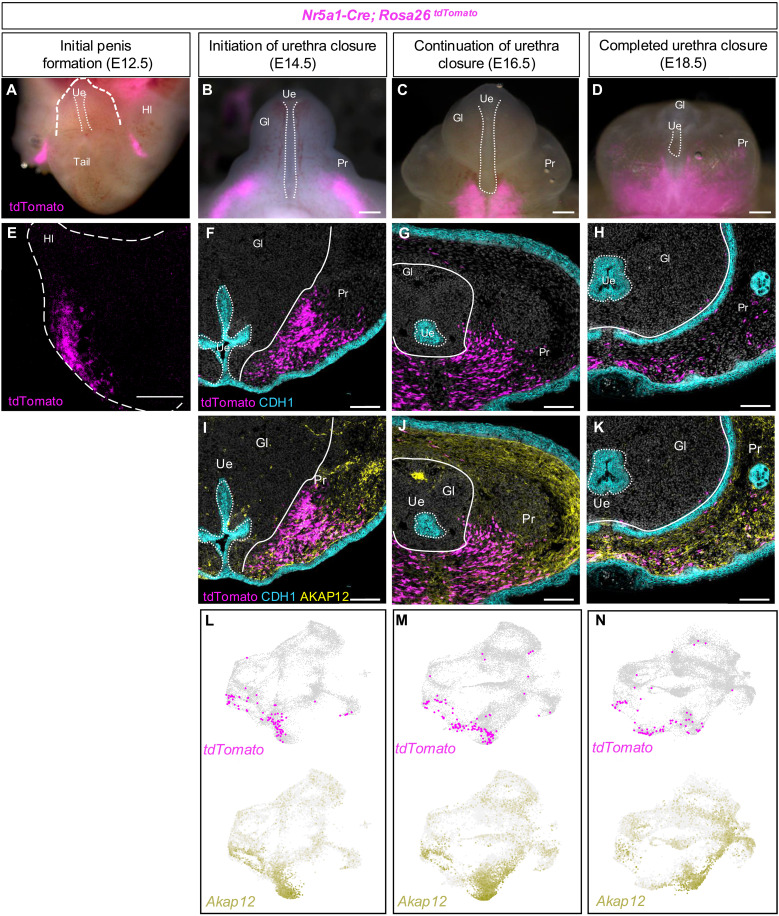
Contribution of hindlimb-derived *Nr5a1^tdTomato+^* cells to the fetal penis. (**A** to **D**) Whole-mount images of external genitalia from E12.5-E18.5 XY mouse embryos. *Nr5a1-Cre*;*Rosa26^tdTomato^* mice were used to label hindlimb cells in magenta. White dotted lines in (A) to (D) mark the open urethra on the ventral aspect of penis. The bracketed line in (A) demarcates the fetal penis. (**E**) Light-sheet image of the right hindlimb of an E12.5 mouse embryo; *Nr5a1^tdTomato+^* cells are labeled in magenta. (**F** to **H**) Immunofluorescent staining of penis cross sections at E14.5, E16.5, and E18.5 with magenta indicating cells with endogenous tdTomato fluorescence and blue labeling the epithelium (CDH1). (**I** to **K**) Overlay of prepuce marker AKAP12 from the same sections in (E) and (F). (**L** to **N**) Single-cell mRNA UMAP displaying the expression of tdTomato (red) and AKAP12 (yellow). Scale bars, [(A) to (E)] 200 μm and [(F) to (K)] 100 μm. Hl, hindlimb; Gl, glans; Pr, prepuce; Ue, urethra.

Sex-specific differentiation of the penis relies on androgens, especially dihydrotestosterone (DHT), for urethra development (*7*, *8*). The enzyme 5α-reductase, expressed in the external genitalia, converts testis-derived testosterone to DHT. Loss-of-function mutations in the 5α-reductase gene (*SRD5A2*) cause severe hypospadias in male individuals (*9*). Despite its role in penis development, the activation mechanism of 5α-reductase in fetal external genitalia remains unclear. We hypothesized that the master regulator of steroidogenesis, NR5A1 (or steroidogenic factor 1), may be responsible for 5α-reductase expression (*10*, *11*). To visualize cells that express *Nr5a1*, we developed the *Nr5a1-Cre^tg/−^*;*Rosa-LSL-tdTomato^+/f^* mouse model where *Nr5a1*^+^ cells are permanently marked with the fluorescent protein tdTomato (or *Nr5a1*^*tdTomato*+^ cells henceforth). We found that, at embryonic day 11.5 (E11.5), 1 day after the onset of penis formation in XY mouse embryos, no *Nr5a1*^*tdTomato*+^ cells were observed in any part of the posterior embryo including the external genitalia (fig. S1). However, 1 day later at E12.5, bilateral clusters of *Nr5a1*^*tdTomato*+^ cells appeared posterior to the hindlimbs in the inguinal region outside of the penis (Fig. 1A). At E14.5, the *Nr5a1^tdTomato+^* cell clusters extended from the inguinal region to bilateral posterior sides of the penis (Fig. 1B). As the urethra continued to close (E16.5 to E18.5), the *Nr5a1^tdTomato+^* cells moved medially and eventually became confluent along the proximal-ventral aspect of the penis (Fig. 1, C and D).

To investigate the identity and location of *Nr5a1^tdTomato+^* cells, we performed immunofluorescence for tdTomato, CDH1 (a marker for the epithelium of the urethra and epidermis), and AKAP12 (a marker for the general prepuce). In the whole-mount E12.5 XY embryos, *Nr5a1*^*tdTomato*+^ cells were not present in the external genitalia and, instead, were found under the epidermis of the inguinal region of the embryo, consistent with the whole-mount images (Fig. 1E and movie S1). At E14.5 onward, the *Nr5a1*^*tdTomato*+^ cells became a part of the preputial swellings based on the partial colocalization of the *Nr5a1^tdTomato^* fluorescence with the general prepuce marker, AKAP12, in both the histological sections and single-cell mRNA sequencing data (Fig. 1, I to N, and fig. S2). At E14.5 and E16.5, ~76 and 65% (*P* < 0.05) of *Nr5a1^tdTomato^* cells, respectively, expressed *Akap12* (*n* = 2)*.* By E18.5, 30% of *Nr5a1^tdTomato^* cells expressed *Akap12* (*n* = 2, *P* > 0.05), suggesting that *Nr5a1^tdTomato^* cells are heterogeneous and diverge from the general prepuce cell population. Throughout the rest of embryonic development, the *Nr5a1*^*tdTomato*+^ cells remained in the prepuce and became a confluent cell population along the ventral aspect of the penis, where the urethra once was located (Fig. 1, F to K). *Nr5a1^tdTomato+^* cells expressed distinct sets of genes, including *Myocd*, *Grem1*, *Scube2*, and others (fig. S3). These genes are commonly associated with smooth muscle cell types.

Although the *Nr5a1-Cre^tg/+^*;*Rosa-LSL-tdTomato^+/f^* model permanently labeled the cells as soon as they expressed *Nr5a1*, such model was unable to inform us the actual expression of *Nr5a1* after the labeling. To profile active expression of *Nr5a1* and determine whether *Nr5a1* is expressed in the genitalia, we examined the external genitalia using an additional *Nr5a1* reporter, *Nr5a1-GFP*, which represent actual *Nr5a1* expression at the time of examination (*12*)*.* An identical pattern of *Nr5a1^tdTomato+^* cell population was observed at E12.5 outside of the penis (fig. S4A). However, in contrast to the *Nr5a1-Cre^tg/+^*;*Rosa-LSL-tdTomato^+/f^* lineage tracing model, *Nr5a1-GFP* expression was not found within the penis at any stage after E13.5 (fig. S4B). This observation was also corroborated by our published single-cell mRNA sequencing dataset, which showed that *Nr5a1* gene expression was only detected in few cells (*2*–*5*) throughout penis development (fig. S4C). We next investigated whether *Nr5a1* is functionally important for penis development by controlling the expression of *Srd5a2*. We developed a conditional knockout of *Nr5a1* with the *Isl1^cre/+^* mice that target hindlimb and external genitalia (*13*). We first examined the *Isl1-Cre* activity by crossing the *Isl1^cre/+^* mice to the *Rosa-tdTomato* reporter mice. We found that the *Isl1-Cre* activity occurred at E10.5 before *Nr5a1* expression in the hindlimbs of the embryo (fig. S5, A and B). By postnatal day 0 (P0), almost the entire penis was positive for *Isl1-Cre* activity (fig. S5, C and D), indicating its feasibility to target *Nr5a1* deletion. When the *Nr5a1* gene was inactivated in the hindlimbs and genitalia (*Isl1^cre/+^*;*Nr5a1^f/−^*), the penis developed normally and *Srd5a2* expression was unchanged (*n* = 3) (fig. S6, A to D). On the basis of in situ hybridization, *Nr5a1^tdTomato+^* cells had minimal expression of *Srd5a2* in wild-type animals (*n* = 3) (fig. S6, A to D)*.* Together, these data reveal that *Nr5a1* is a lineage identifier that transiently marks a distinct extragenital progenitor population derived from outside of the embryonic penis. The expression of *Nr5a1* did not have an impact on urethra closure nor *Srd5a2* expression.

### The *Nr5a1*^*tdTomato+*^ cells actively migrate during urethra closure

**Fig. 2.**
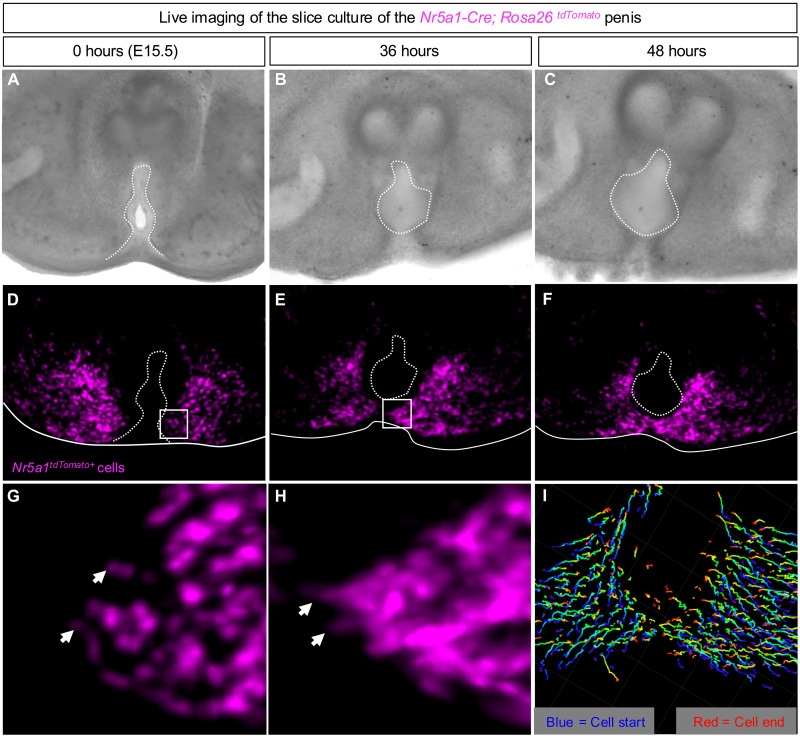
*Nr5a1^tdTomato+^* cells undergo extensive and active migration during urethra closure. (**A** to **C**) Bright-field images of penis slice cultures from *Nr5a1-Cre*;*Rosa26^tdTomato^* embryos at 0, 36, and 48 hours. (**D** to **F**) Fluorescent images of penis slice cultures from Nr5a1-Cre;*Rosa26^tdTomato^* embryos. Dotted lines represent the urethra, and solid lines outline the penis slice. White boxes in (D) and (F) represent magnified regions in (G) and (H)*.* White arrowheads designate filopodia in the same cell at 0 and 36 hours. (**I**) Dragon tail tracks generated by Imaris to mark the cell migratory paths. Blue colors represent the cell position at 0 hours, and red color is the position of the cells at 48 hours.

To visualize the movement of the *Nr5a1*^*tdTomato*+^ cells during urethra closure, we developed an ex vivo penis slice culture system that enables us to observe the movement of *Nr5a1*^*tdTomato*+^ cells in real time (*14*). We collected external genitalia at E15.5, when urethra closure starts, and sliced them into 150-μm-thick sections for live imaging. At the beginning of the culture (0 hours), the urethra was physically connected to the skin and was not a closed tube in the center of the penis (Fig. 2A). The *Nr5a1*^*tdTomato*+^ cells at this time showed few signs of active cell migration (a lack of visible lamellipodia) and were bilateral to the central urethra (Fig. 2, A and D, and movie S2). Thirty-six hours after culture, lamellipodia were observed at the leading edge of the *Nr5a1*^*tdTomato*+^ cell population, and the distance between the bilateral populations of *Nr5a1*^*tdTomato*+^ cells shortened (Fig. 2, B and E, and movie S2). By 48 hours, the *Nr5a1*^*tdTomato*+^ cells filled in the space ventrally where the urethra was once open, resulting in a closed urethra (77% of experimental samples, *n* = 9) (Fig. 2C and movie S2). Upon investigating the migration tracks using the Imaris software, we found that the majority of the *Nr5a1*^*tdTomato*+^ cells migrate medially toward the urethra (Fig. 2F).

### Loss of *Nr5a1*^*tdTomato+*^ cells results in severe hypospadias

**Fig. 3.**
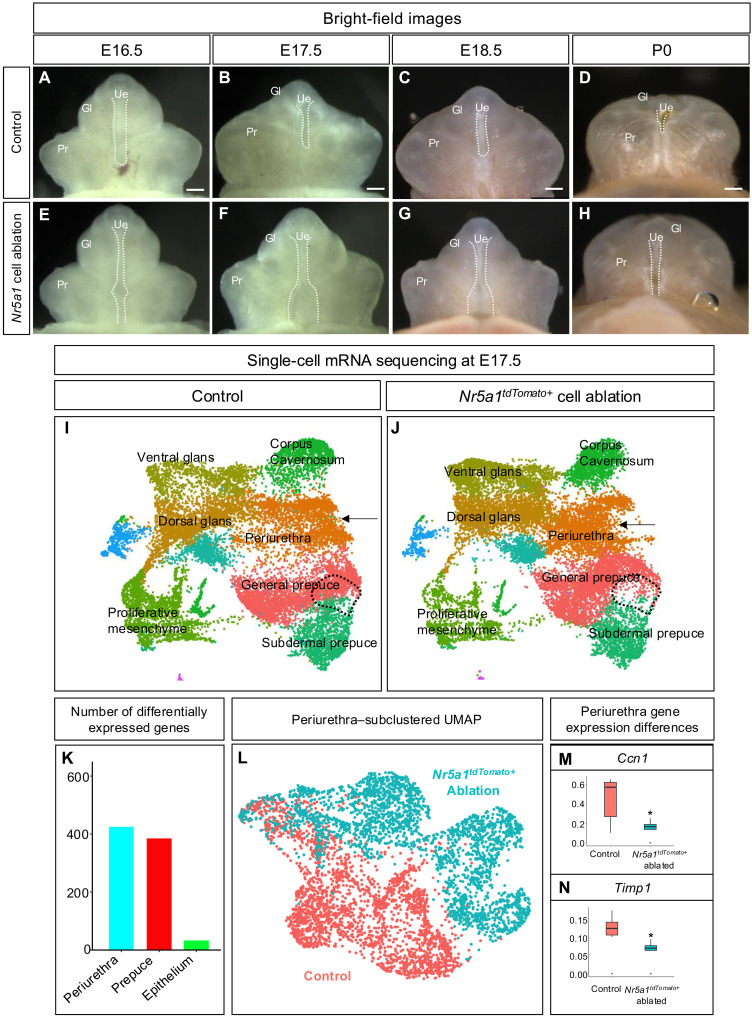
*Nr5a1^tdTomato+^* cells are required for urethra closure. (**A** to **H**) Whole-mount images of control [(A) to (D)] and *Nr5a1^tdTomato+^* cell–ablated [(E) to (H)] penises at E16.5, E17.5, E18.5, and P0. Dotted lines display regions where the urethra is open. (**I** and **J**) UMAP of control (I) and *Nr5a1^tdTomato+^* cell–ablated (J) penises with colors representing unbiased clusters of cells. Black dotted line represents a region where *Nr5a1^tdTomato+^* cells are lost in *Nr5a1^tdTomato+^* ablated penises, and black arrows represent a shift in periurethra transcriptome. (**K**) Bar graph of differentially regulated genes in the periurethra, prepuce, and epithelium. (**L**) Re-clustered UMAP of periurethral cells from control (red) and *Nr5a1^tdTomato+^* cell–ablated penises (blue). (**M** and **N**) Significantly altered genes (Ccn1 is a cell migration–related gene, and Timp1 is an extracellular matrix modifier) in *Nr5a1^tdTomato+^* cell–ablated periurethra. *P < 0.05.

To test whether the *Nr5a1*^*tdTomato*+^ cells are essential for urethra closure, we ablated these cells using the diphtheria toxin cell ablation or *Rosa-LSL-DTA* model (*15*). In the presence of *Nr5a1-Cre*^*Tg*/+^, diphtheria toxin or DTA was induced, which halted translation and eventually caused cell death of the *Nr5a1*^*tdTomato*+^ cells. Ablation of *Nr5a1*^*tdTomato*+^ cells disrupted urethral closure as early as E16.5 (Fig. 3, A and E). At E17.5, when urethra closure had further progressed in the control embryos, the urethra of the *Nr5a1^tdTomato+^* cell–ablated mice remained open (Fig. 3, B and F). By E18.5 and P0, hypospadias was clearly apparent in the *Nr5a1*^*tdTomato*+^ cell–ablated mice (*n* = 7, 100% of mice) (Fig. 3, C, D, G, and H). Invariably, all *Nr5a1*^*tdTomato*+^ cell–ablated male mice developed extremely severe cases of hypospadias and preputial hypoplasia (Fig. 3, G and H). Histological sections demonstrated that the urethra was open at the base of the *Nr5a1*^*tdTomato*+^ cell–ablated penis in contrast to the control male where the urethra was completely closed (fig. S7, A and B). We further confirmed that *Nr5a1^tdTomato+^* cell ablation sufficiently removed the majority of the *Nr5a1^tdTomato+^* cells in these hypospadias penises (fig. S7, C and D). After conducting cell counts on the number of *Nr5a1^tdTomato+^* cells per section, we found a 72% decrease of *Nr5a1^tdTomato+^* cells in DTA^+^ penises (*P* < 0.05, *n* = 5). Overall, our results demonstrate that ablation of *Nr5a1^tdTomato+^* cells resulted in severe hypospadias.

The *Nr5a1-Cre*–induced ablation occurred in not only the penis but also the testes and adrenals. The size of the testes in *Nr5a1^tdTomato+^* cell–ablated embryos was markedly reduced and the adrenal glands could not be found in any of the embryos, demonstrating the efficiency of the cell ablation model (fig. S8, A to C). However, without functional testes, secondary impacts on the androgen-responsive organs, such as the external genitalia, are possible. To exclude the possibility that the hypospadias phenotypes in the *Nr5a1^tdTomato+^* cell–ablated penis were secondary to the degenerated testes and consequent androgen insufficiency, we supplemented the *Nr5a1^tdTomato+^;Rosa-DTA* embryos with testosterone propionate (0.2 mg per 10 g of body weight) during the critical window of urethra closure (E13.5 to E18.5) by injecting pregnant dams. The dose of testosterone was based on previously published papers (*16*), which was sufficient to masculinize female embryos, using androgen-controlled anogenital distance as the indicator (fig. S7, D and E). The *Nr5a1^tdTomato+^* cell–ablated male embryos that were exposed to testosterone propionate still developed severe cases of hypospadias in 75% of the animals (*n* = 7, *P* < 0.05) (fig. S7D). These data indicate that some of the hypospadias phenotype could be due to the loss of testis-derived androgens, but the loss of *Nr5a1^tdTomato+^* cells is the major cause of severe hypospadias.

Next, we investigated how the loss of *Nr5a1*^*tdTomato*+^ cells influences cell population dynamics in the penis. We conducted single-cell mRNA sequencing on control (*Nr5a1-Cre^+/+^;RosaDTA^+/f^*) and *Nr5a1*^*tdTomato*+^ cell–ablated (*Nr5a1-Cre^Tg/+^;RosaDTA^+/f^*) penises at the time of urethra closure (*n* = 2) (E16.5). The unbiased cell clusters, or the composition of the cell populations, were similar between the control and *Nr5a1*^*tdTomato*+^ cell–ablated penis; however, transcriptomic shifts in several cell populations were identified (Fig. 3, I and J). Markers of the *Nr5a1^tdTomato+^* cell population, *Grem1* and *Scube2*, were significantly down-regulated, confirming the loss of *Nr5a1^tdTomato+^* cells (fig. S8, A and B). Upon investigating the number of differentially expressed genes between the control and *Nr5a1^tdTomato+^* cell–ablated samples for each penis cell population, we found that the corpus cavernosum, periurethra, ventral distal glans, preputial mesenchyme, dorsal distal glans, and subdermal prepuce had the first (637), second (425), third (422), fourth (385), fifth (336), and sixth (217) most changed genes as a result of loss of *Nr5a1*^*tdTomato*+^ cells (Fig. 3K and fig. S9A). The urethra epithelium had 33 genes differentially expressed and was one of the cell populations with the least amount of gene expression change due to *Nr5a1^tdTomato+^* cell ablation (Fig. 3K and fig. S9A). These data indicate that removal of *Nr5a1*^*tdTomato*+^ cells has a global and yet cell-type–specific impact on the cell populations of the external genitalia.

To gain insight into how the periurethral cells were affected in the absence of *Nr5a1*^*tdTomato*+^ cells, we bioinformatically separated these cells from the single-cell dataset (Fig. 3, I and J) and re-clustered them (Fig. 3L). The periurethral cells in the *Nr5a1*^*tdTomato*+^ cell–ablated penis were significantly different from the control in their transcriptome with 654 differentially expressed genes (Fig. 3L). Gene Ontology analysis on the differentially expressed genes showed significant changes in pathways such as axonal guidance and fibrosis pathways (fig. S9B). In the axonal guidance pathways, several cell migration–related genes (*Cnn1*, *Dner*, and others) were altered, while the fibrotic pathways had changes in extracellular matrix gene expression (*Timp1*, *Itga4*, and others) (Fig. 3, M and N, and fig. S9C). The loss of *Nr5a1*^*tdTomato*+^ cells has a substantial impact on the periurethral cells, particularly the expression of extracellular matrix and cell migration genes in these cells.

### *Nr5a1^tdTomato+^* cells interact extensively with the neighboring periurethra mesenchyme

**Fig. 4.**
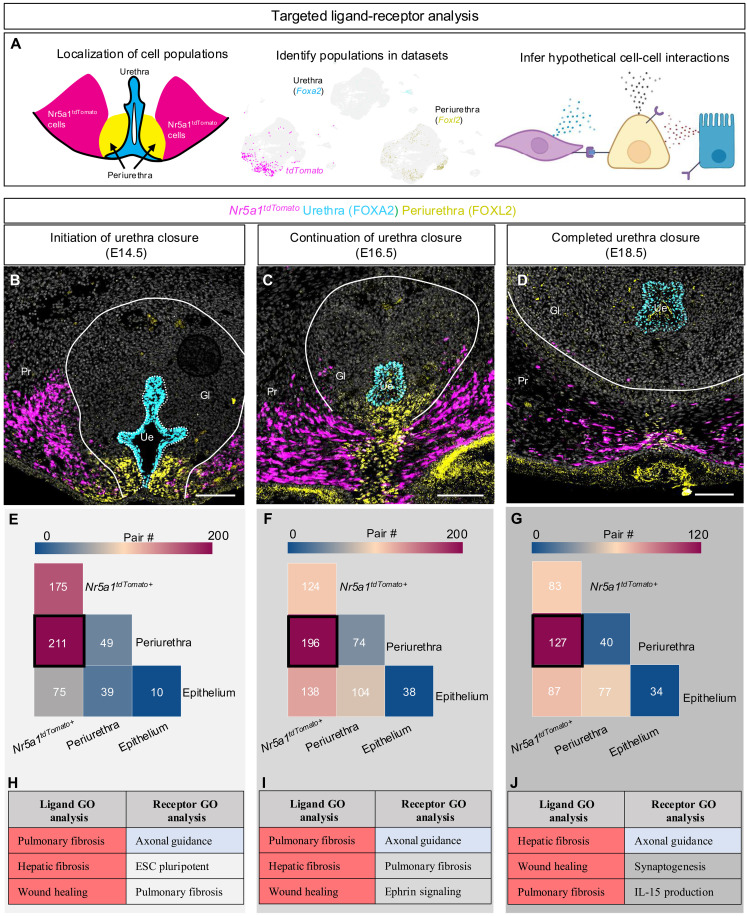
*Nr5a1^tdTomato+^* cells communicate extensively with neighboring mesenchymal cells. (**A**) Depiction of experimental design to identify potential ligand-receptor interactions. (**B** to **D**) Immunofluorescence of coronal penis sections at E14.5, E16.5, and E18.5, with FOXA2 (cyan for urethra), FOXL2 (yellow for periurethra), and tdTomato (magenta for *Nr5a1^tdTomato+^* cells). Scale bars, 100 μm. Gl, glans; Pr, prepuce; and Ue, urethra. (**E** to **G**) Tile plots were used to present the numbers of ligand-receptor pairs with red representing a high number of ligand-receptor pairs and blue representing a low number of ligand-receptor pairs. (**H** to **J**) Gene Ontology (GO) analysis of ligands and receptors identified from the ligand-receptor analysis within the periurethral cell population. Terms are listed in order of significance. Magenta-shaded cells represent conserved processes for ligands, blue-shaded cells represent conserved ontologies for receptors, and gray-shaded cells represent ontologies not conserved between all stages. IL-15, interleukin-15. ESC, embryonic stem cell.

To investigate how *Nr5a1*^*tdTomato*+^ cells communicate with the urethra epithelium and periurethra mesenchyme, we identified potential ligands and receptors expressed by these three distinct cell types over the course of urethra closure by analyzing the single-cell sequencing data with the CellPhoneDB v2.0 package (Fig. 4A) (*17*). At the initiation of urethra closure (E14.5), extensive potential interactions (211 ligand-receptor pairs) were detected between the *Nr5a1*^*tdTomato*+^ cells (magenta) and the periurethra (yellow) mesenchyme (Fig. 4, B and E). Interactions between the epithelium (cyan) and either the periurethra or *Nr5a1*^*tdTomato*+^ cells were low to moderate (Fig. 4, B and E). Gene Ontology analyses of the ligands and receptor pairs between the *Nr5a1*^*tdTomato*+^ cells and periurethra revealed a significant enrichment of fibrotic and cell migration–related pathways. Similar relationships were found at both the continuation of urethra closure at E16.5 and completed urethra closure stages at E18.5 (Fig. 4, F, G, I, and J). At these two stages, interaction between *Nr5a1*^*tdTomato*+^ and periurethral cells remained to be the strongest, although the number of ligand-receptor interactions decreased at E18.5 (*n* = 127) (Fig. 4, E to G). Results from these analyses reveal that there is extensive communication between the *Nr5a1*^*tdTomato*+^ cells and the neighboring periurethra and a moderate amount of interaction between *Nr5a1*^*tdTomato*+^ cells and urethral epithelial cells.

### *Nr5a1*^*tdTomato+*^ cell migration and urethra closure are dependent on EGF signaling

**Fig. 5.**
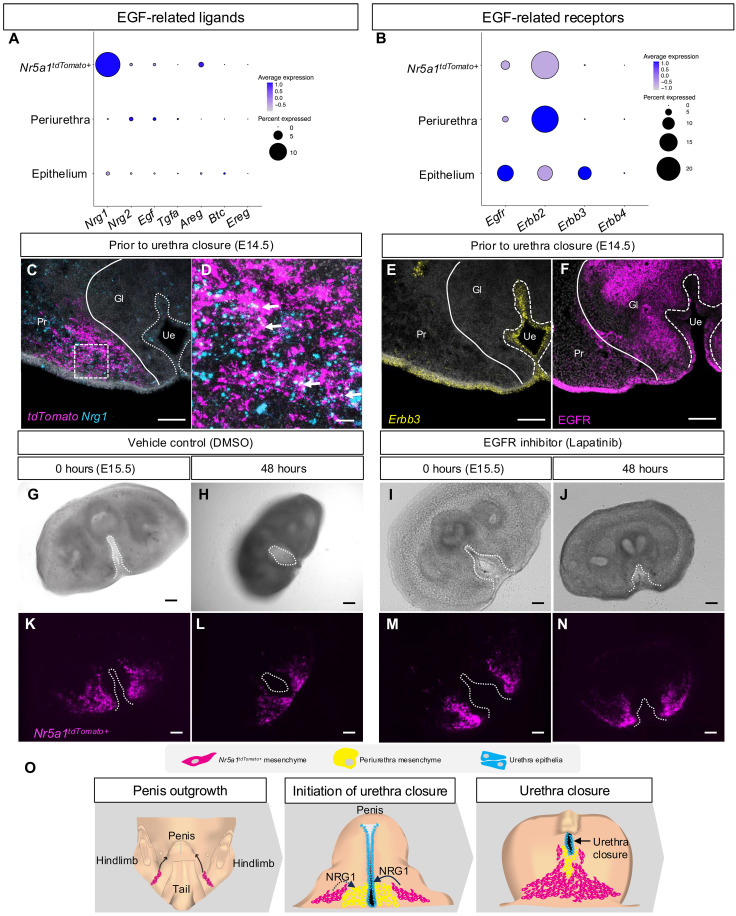
EGF signaling from *Nr5a1^tdTomato+^* cells as a critical pathway for urethra closure. (**A** and **B**) Dot plot of EGF-related ligand and receptor gene expression from single-cell mRNA sequencing data of normal genitalia at E14.5. (**C** and **D**) Low and high magnifications of RNAscope images of control penis sections at E14.5 stained for *Nrg1* (cyan) and tdTomato (magenta). (**E** and **F**) RNAscope and immunofluorescent images of control penis sections for *Erbb3* (yellow) and EGFR (magenta) (**G** to **J**) Bright-field and (**K** to **N**) fluorescent images of control and EGFR inhibitor exposed slice culture sections at 0 and 48 hours. (**O**) Summary of *Nr5a1^tdTomato+^* cell role in penis development. White dotted lines outline the urethra in all images. Scale bars, [(B) and (E) to (N)] 100 μm and (C) 20 μm. Gl, glans; Pr, prepuce; Ue, urethra.

To identify the mechanism through which *Nr5a1*^*tdTomato*+^ cells facilitate urethra closure, we searched the CellPhoneDB results for critical paracrine signaling factors from *Nr5a1*^*tdTomato*+^ cells (*18*). *Neuregulin-1* (*Nrg1*), a member of the epidermal growth factor (EGF) family, was significantly enriched in the *Nr5a1*^*tdTomato*+^ cells compared to periurethra mesenchyme and urethra epithelium just before the initiation of urethra closure (E14.5) (Fig. 5A). Other EGF members (*Egf*, *Tgfa*, *Areg*, *Btc*, and *Ereg*) had little to no expression in most of the cell populations (Fig. 5A). Using RNAscope, we found considerable enrichment of *Nrg1* mRNA in the *Nr5a1*^*tdTomato*+^ cells in the penis (Fig. 5, C and D), supporting the single-cell mRNA sequencing data. NRG1 proteins signal through the receptor tyrosine kinases ERBB3 and ERBB4 and with less affinity to EGF receptor (EGFR) in several cell types (*19*). ERBB2 has no known ligand and mainly serves as a critical co-regulator for the other three EGF-related receptors (*20*). The periurethra mesenchyme had significant enrichment of *Egfr* and *Erbb2* receptors, whereas the urethra epithelium expressed *Egfr*, *Erbb2*, and *Erbb3* (Fig. 5B). The *Nr5a1*^*tdTomato*+^ cells had some enrichment of *Egfr* and *Erbb2*; however, expression intensity of these genes was low. These data implicate both the periurethra and urethra epithelium as potential targets of *Nr5a1*^*tdTomato*+^ cell–derived NRG1.

To determine whether EGF signaling through *Nr5a1^tdTomato+^* cell–derived NRG1 is essential for urethra closure, we took advantage of the slice culture system that we developed (Fig. 2) and cultured the *Nr5a1^tdTomato+^* penis slices with Lapatinib, an EGFR and ERBB inhibitor, or dimethyl sulfoxide (DMSO) as the vehicle control (Fig. 5, G to N). Lapatinib specifically inhibits the phosphorylation and activation of the tyrosine kinases, EGFR and ERBB2 (*21*, *22*). Penis slices exposed to DHT and DMSO underwent normal urethra closure with the *Nr5a1*^*tdTomato*+^ cells, completing full migration (75% urethra closure, *n* = 6) (Fig. 5, G, H, K, and L, and movie S3). On the other hand, DHT and Lapatinib inhibited *Nr5a1*^*tdTomato*+^ cell migration and led to failure of urethra closure (0% urethra closure, *n* = 7) (Fig. 5, I, J, M, and N, and movie S4). These data support that the EGF signaling, likely activated by NRG1 from the *Nr5a1*^+^ mesenchymal cells, facilitates urethra closure.

## DISCUSSION

Formation of the penis requires the coordination of cells from unique developmental origins. Cells derived from the umbilicus and the tailgut undergo extensive outgrowth and differentiation to form the dorsal and ventral aspects of the genital tubercle, respectively. Here, we identify that a transcriptionally distinct group of cells, originating from the inguinal region of the embryo, contributes to penis development, particularly the closure of urethra (Fig. 5O). Once these extragenital cells enter the penis, they undergo extensive migration and secrete paracrine factors that facilitate urethra closure (Fig. 5O).

Cells derived from the inguinal region have been suggested to be involved in penis development through evolutionary studies and correlations of human genital defects with hindlimb abnormalities. In chickens, cells posterior to the hindlimb had the potential to occupy the genital tubercle (*23*). The hemipenes in squamate reptiles, which is a paired genital organ, form immediately adjacent to the hindlimb buds of the embryo (*24*, *25*). Hypospadias and other genital defects in humans were correlated with hindlimb abnormalities. For example, cases of sirenomelia, a birth defect in humans where the hindlimbs fuse together during development, are associated with hypospadias and penile hypoplasia (*26*, *27*). In mice, loss of *Bmp4*, *Wnt5a*, or *Hoxa13* caused disruptions in both hindlimb and penis development (*26*, *28*, *29*). This circumstantial evidence points to the potential contribution of extragenital cells near the hindlimb to penis formation. However, this hypothesis has never been definitively tested in a mammalian organism. We discover that, in the mouse embryo, the extragenital cells originate near the inguinal region of the embryo and then migrate to the developing penis.

The extragenital cells, but not other cell populations in the penis, have enriched expression of a ligand from the EGF signaling family, *Nrg1*. NRG1 is commonly associated with cell migration in Schwann cells and neural crest cells (*22*). In intestinal organoids of epithelial cells, NRG1 treatment induced actin-cytoskeleton alternations, which caused increased budding and tube formation (*19*, *30*). NRG1 signals mainly through receptors ERBB3 and ERBB4, which commonly heterodimerize with EGFR and ERBB2 to facilitate signaling (*31*). *Egfr* and *Erbb3* were expressed in the periurethral cells and adjacent urethra epithelium, suggesting that NRG1 from the extragenital cells could act as a paracrine factor on either the periurethral cells or urethra epithelium to aid in urethra closure.

In addition to being enriched with signaling ligands, the *Nr5a1^tdTomato+^* cells were enriched with a suite of extracellular matrix modifiers (*Timp1*, *Adamts9*, *Col23a1*, *Col6a6*, and others). Also, a large portion of the periurethra/urethra/*Nr5a1^tdTomato+^* communication was related to extracellular matrix components. Extracellular matrix components are critical for tubular formation across organ systems (*32*). In neural tube closure, the proteases, *Adamts9* and *Adamts20*, are critical for proper tubular formation (*33*). When *Adamts9* and *Adamts20* are knocked out, versican, a component of extracellular matrix, was elevated. In *Pax3*-knockout mice, versican is abundantly expressed, which is correlated with inhibition of neural crest cell migration and the closure of the neural tube (*34*). The developing penis has abundant expression of versican, and modifications of this extracellular matrix protein and others could play a role in urethra closure.

Despite that birth defects of the male urogenital system are some of the most common birth defects in the world, a large proportion of human hypospadias cases remain unexplained. This is likely due to the lack of information on gene regulation and cell population function within the developing penis. With the findings from this study, the genetic and environmental contributors to hypospadias can be better identified, thus allowing us to develop preventatives or better alternative treatment for this far too common birth defect.

## MATERIALS AND METHODS

### Mouse models

*Nr5a1-Cre^Tg/Tg^* [B6D2-*g(Nr5a1-cre)2Klp*] mice were generated by the late Keith Parker (*35*). *Rosa-LSL-tdTomato^f/f^* [B6.Cg-*Gt(ROSA)26Sor ^tm9(CAG-tdTomato)Hze^*], Isl1-Cre^cre/+^ [Isl1tm1(cre)Sev/J], and *Rosa-LSL-DTA^f/f^* [C.129P2(B6)-Gt(ROSA)26Sortm1(DTA)Lky/J] were purchased at the Jackson Laboratory (*36*). All mice used for lineage tracing and single-cell RNA sequencing contain the alleles for *Nr5a1-Cre^Tg/+^;Rosa-LSL-tdTomato^+/f^. Nr5a1-Cre^Tg/+^;Rosa-LSL-tdTomato^+/f^* were generated by crossing *Nr5a1-Cre^Tg/Tg^* male mice with female *Rosa-LSL-tdTomato^f/f^*. Observation of a vaginal plug was defined as embryonic day or E0.5. At dissection developmental stage was confirmed by the Theiler staging criteria, and mice were sexed by evaluating presence of testis or ovaries. Presence of tdTomato fluorescence was used to confirm the genotype.

*Nr5a1^tdTomato+^* cell ablation experiments used a mouse model with a diphtheria toxin gene inserted into the Rosa allele (*Rosa-LSL-DTA^f/f^*) (*15*). To generate *Nr5a1^tdTomato+^* cell ablation mice (*Nr5a1-Cre^Tg/+^;Rosa-LSL-tdTomato^+/f^;Rosa-LSL-DTA^+/f^*), *Nr5a1-Cre^Tg/+^;Rosa-LSL-tdTomato^+/f^* male mice were crossed with *Rosa-LSL-DTA^f/f^* female mice. Embryos were sexed by the presence (female) or absence (male) of the Barr body via Giemsa staining of the amnion (*37*) and were genotyped by probing for the presence of a Cre allele. We confirmed that *Nr5a1-Cre* also targets the adrenal glands and activation of diphtheria toxin caused a complete loss of adrenals (fig. S7). To determine the role of lack of androgens in the phenotype of these *Nr5a1^tdTomato+^* cell ablation embryos, pregnant *Rosa-LSL-DTA^f/f^* females were supplemented daily subcutaneously with 0.2 mg/10 g of testosterone propionate (Sigma-Aldrich, T1875-5G) from E13.5 to E18.5 to replace lost testosterone due to testis ablation. All animal procedures were approved by the National Institute of Environmental Health Sciences (NIEHS) Animal Care and Use Committee and follow a NIEHS-approved animal study proposal (ASP 2010-0016).

### Slice culture of the external genitalia

*Rosa-LSL-tdTomato^f/f^* female mice crossed with *Nr5a1-Cre^Tg/Tg^* male mice were euthanized at E15.5. Once pups were removed from the uterus and decapitated, bodies were kept on ice. Male embryos were separated on the basis of the presence of testis. Female embryos were not used in these studies. Once sex was defined, male genitalia were removed and embedded in 4% low melting agarose (GeneMate E-3112-125). Low melting agarose was mixed with sterile 1× phosphate-buffered saline (PBS) and microwaved for 1 min to dissolve, and it was kept at 37°C for a minimum of 1 hour before embedding. Leica VT1000S vibratome was used to make 150-μm coronal sections of penises. Section speed was between 0.1 and 0.3 mm/s to maintain tissue integrity. All sectioning was conducted in ice cold, sterile 1× PBS. Sections were selected for culture based on (i) presence of tdTomato fluorescent *Nr5a1^tdTomato+^* cells and (ii) lack of urethra closure. The selected sections were placed in a 0.4-μm cell culture insert (MilliporeSigma, PICM01250) that was saturated with slice culture medium. Slice culture medium consisted of phenol red–free Dulbecco’s modified Eagle’s medium/F12 (Gibco, 21041-025), 10% charcoal-stripped fetal bovine serum (Sigma-Aldrich, F6765), and 1% penicillin/streptomycin (Sigma-Aldrich, P4333). DHT (10^−4^ M) (Steraloids, A2570-011) or Lapatinib (1 μM) (Sigma-Aldrich, D8418-100ML) was added to examine the loss of EGF-related signaling. Culture inserts were placed inside either a 35-mm glass bottom petri dish (Ted Pella, 14022-1120) or a 12-well glass bottom culture plate (Cellvis, P12-1.5H-N) with 1 ml of slice culture medium. Petri dish was placed into Keyence BZ-X810 for live cell imaging. Images were taken at 10-min intervals with a 10-μm *z*-stack with a 10× objective. Imaging lasted for at least 48 hours to capture urethra closure. The captured images were compiled into movies with the Keyence BX-X800 analyzer. To create cell tracks, movies were imported into Imaris, and the Dragon tail feature was applied to red fluorescing cells. All experiments involving slice culture were conducted at least four separate times.

### RNAscope, immunofluorescence, and light-sheet imaging

Penises were fixed in 4% paraformaldehyde overnight at 4°C, watched in 20% sucrose in 1× PBS, embedded in optimal cutting temperature compound, and sectioned at 10 μm. Sections were mounted on Superfrost Plus slides (Fisherbrand, 12–550-15) and kept at −80°C until further processing. For RNAscope probes, *Scube2* (ACD 488141), *Nrg1* (ACD 418181-C3), *Erbb3* (ACD 441801-C2), *Grem1* (ACD 314741-C3), *tdTomato* (ACD 317041 and 317041-C2), and *Srd5a2* (ACD 431361) were used according the ACDBio’s protocol. For immunofluorescence, sections were permeabilized in 1× PBS with 0.1% Triton X-100 and blocked for 1 hour with 10% donkey serum. Primary antibodies against AKAP12 (gift from I. H. Gelman, Roswell Park Cancer Institute), dsRED (Clontech, 632496), FOXL2 (Abcam, ab5096), EGFR (Abcam, ab32077), and FOXA1 (Abcam, ab170933) (table S2) were incubated on slides at 4°C overnight. After incubations, slides were washed with 1% Triton, and secondary antibodies (Alexa Fluor 488, 594, and 647) were incubated for 2 hours at room temperature. Slides were counterstained with 4′,6-diamidino-2-phenylindole (1:1000) (MilliporeSigma, 5.08741.0001), were mounted with ProLong Antifade (Invitrogen, P36970), and were imaged on a Zeiss 900 confocal microscope the day after processing. All RNAscope studies were conducted on at least three separate pups.

For light-sheet imaging, samples were fixed in in 4% paraformaldehyde overnight at 4°C and then cleared by strictly using the iDISCO protocol (*38*). Briefly, samples were dehydrated with a methanol series and then bleached with 3% H_2_O_2_, delipidated with 66% dichloromethane, and rehydrated with a methanol series. Samples were then permeabilized, blocked, and labeled with dsRED (Clontech, 632496) and an anti-rabbit Alexa Fluor 647 secondary antibody. Last, the samples were dehydrated in a methanol series, delipidated with dicholormethane, and cleared in dibenzyl ether. Samples were image on the LaVision ultramicroscope II at University of North Carolina at Chapel Hill. Images were processed with Imaris. Two individual samples were cleared and processed with light-sheet microscopy.

### Cell disassociation and single-cell suspension

Preparation for single-cell sequencing follows the same protocol in our published paper (*18*). Penises from E16.5 embryos (*n* = 2) were placed in 250 μl of ice-cold disassociation medium [sterilized 1× PBS and 0.04% bovine serum albumin, dispase II (1.2 U/ml; Roche 04942078001), collagenase IV (1 mg/ml; Gibco, 17104–019), and deoxyribonuclease I (5 U/ml; Roche 1010459001)]. After the tissues were successfully homogenized, the cells were washed with 1× PBS with 0.04% bovine serum albumin. Cells were loaded into the 10x Chromium machine for single-cell separation.

### Library preparation, sequencing, and sequence alignments

Data from GSE174712 were used for the initial characterization of the *Nr5a1^tdTomato+^* cells (*n* = 2) (*18*). For the cell ablation model, library preparation was conducted following the 10x Genomics library preparation protocol on two replicates for control and DTA^+^ mice. The libraries were pooled into a single tube and sequenced on an Illumina NovaSeq 6000 with an S2 flow cell. Paired-end sequencing was conducted with the first read being 26 base pairs (bp) and the second read being 96 bp. DTA libraries were sequenced to at least 13,000 total reads per cell (table S1), which was sufficient to obtain clustering and differential gene expression analysis. Both library preparation and sequencing were performed by the Epigenomics and DNA Sequencing Core Facility at NIEHS. To detect *tdTomato* mRNA transcripts in the single-cell sequencing data, we added the *tdTomato* coding sequence to the mm10 genome and genes files. Cellranger v3 was then used to align the data with the *tdTomato* sequence.

### Single-cell data analysis

Aligned and annotated datasets were imported into R for Seurat analysis (v. 4.2.1) (*39*). Cells with <7500 and >1000 mRNA counts were removed from the dataset. Cells with mitochondrial content of >25%, Hba content of >25%, and Hbb content of >50% were removed from the datasets. Log normalization, data scaling, dimensionality reduction, and clustering were conducted both the cell ablation and developmental series datasets. Once data were processed, we tested whether *tdTomato* could be properly detected. After plotting a UMAP of *tdTomato* gene expression, it was apparent that the transcript expression was leaky as previously reported by (*40*), although leaky transcript expression did not result in red fluorescent protein in all genitalia cells. There was a low background level of expression throughout most cells. Although most cells did express *tdTomato*, there was a clear separation of high- or low-tdTomato–expressing cells. Thus, cells with <10 counts of tdTomato expression were considered *Nr5a1^tdTomato^* negative, and cells with >10 counts were *Nr5a1^tdTomato^* positive. To validate that *Nr5a1^tdTomato^*-positive cells truly represented the cells identified within the mouse penis, we identified genes that were significantly enriched in the *Nr5a1^tdTomato^-*positive cells and conducted RNAscope for markers *Grem1*, *Scube2*, and *Nrg1.* Once *Nr5a1^tdTomato^*-positive cells were correctly identified, the developmental series dataset was used to investigate ligand-receptor interactions. Epithelial, periurethra, and *Nr5a1^tdTomato+^* cells were subsetted into a separate dataset, and ligand-receptor analysis was executed with Cellphone DB v2.0 (*17*).

The cell ablation dataset was processed the same as the developmental series data. To identify differential expression between control and *Nr5a1^tdTomato^* cell–ablated mice, likelihood ratio tests were conducted between the genotypes for each cell population. To further investigate the changes within the periurethra, that cell population was subsetted, re-clustered, and visualized with a Uniform Manifold Approximation and Projection (UMAP).

### Statistical analysis

For individual gene expression analysis, anogenital measurement comparisons, and hypospadias severity comparisons, linear mixed-effect models were used to calculate statistical differences (*P* < 0.05) (*41*). For single-cell gene expression analysis, each biological replicate was treated as the random effect. For anogenital distance and hypospadias severity comparisons, dam was classified was the random effect.
