# Supplementary material for: An extragenital cell population contributes to urethra closure during mouse penis development
Source: Sci Adv. 2024 Dec 6;10(49):eadp0673. doi: 10.1126/sciadv.adp0673 (PMC11623300; doi:10.1126/sciadv.adp0673)
Supplement: Supplementary file 1 — Legends for movies S1 to S4 Figs. S1 to S10 Tables S1 and S2 [file sciadv.adp0673_sm.pdf]

Science Advances
AAAS

Supplementary Materials for

An extragenital cell population contributes to urethra closure during mouse penis development

Ciro Maurizio Amato *et al*.

Corresponding author: Humphrey Hung-Chang Yao, humphrey.yao@nih.gov

Sci. Adv. **10**, eadp0673 (2024)
DOI: 10.1126/sciadv.adp0673

The PDF file includes:

Legends for movies S1 to S4
Figs. S1 to S10
Tables S1 and S2

Other Supplementary Material for this manuscript includes the following:

Movies S1 to S4

Supplemental Movies

Movie S1. **Lightsheet z-stack of *Nr5a1*$^{tdTomato+}$ cells in E12.5 embryo**

Movie S2. ***Nr5a1*$^{tdTomato+}$ cell migration during urethra closure**

Movie S3. ***Nr5a1*$^{tdTomato+}$ cell migration during urethra closure in control slice**

Movie S4. ***Nr5a1*$^{tdTomato+}$ cell migration during urethra closure in lapatinib exposed slice**

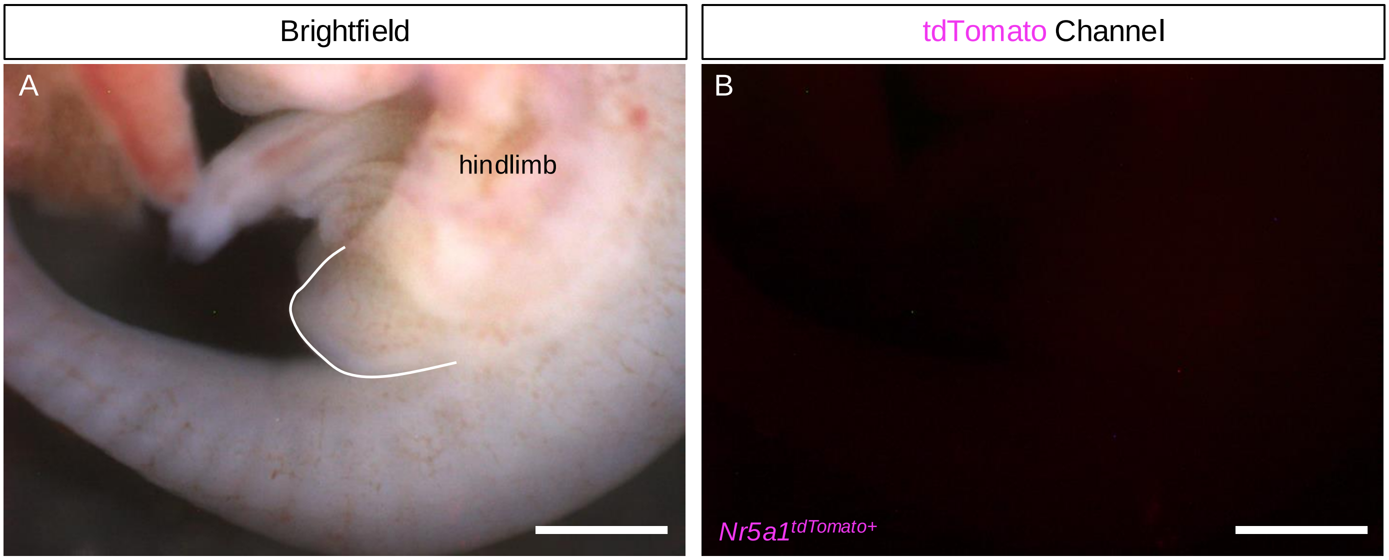
Brightfield
tdTomato Channel
A
hindlimb
B
Nr5a1tdTomato+

Supplemental Figure Legends

Figure S1. *Nr5a1*$^{tdTomato+}$ cells are not found near hindlimbs at E11.5. (A) Brightfield images of E11.5 embryo with the solid white line outlining the genitalia. (B) Fluorescent image of the same E11.5 embryo. Scale bar = 500 µm

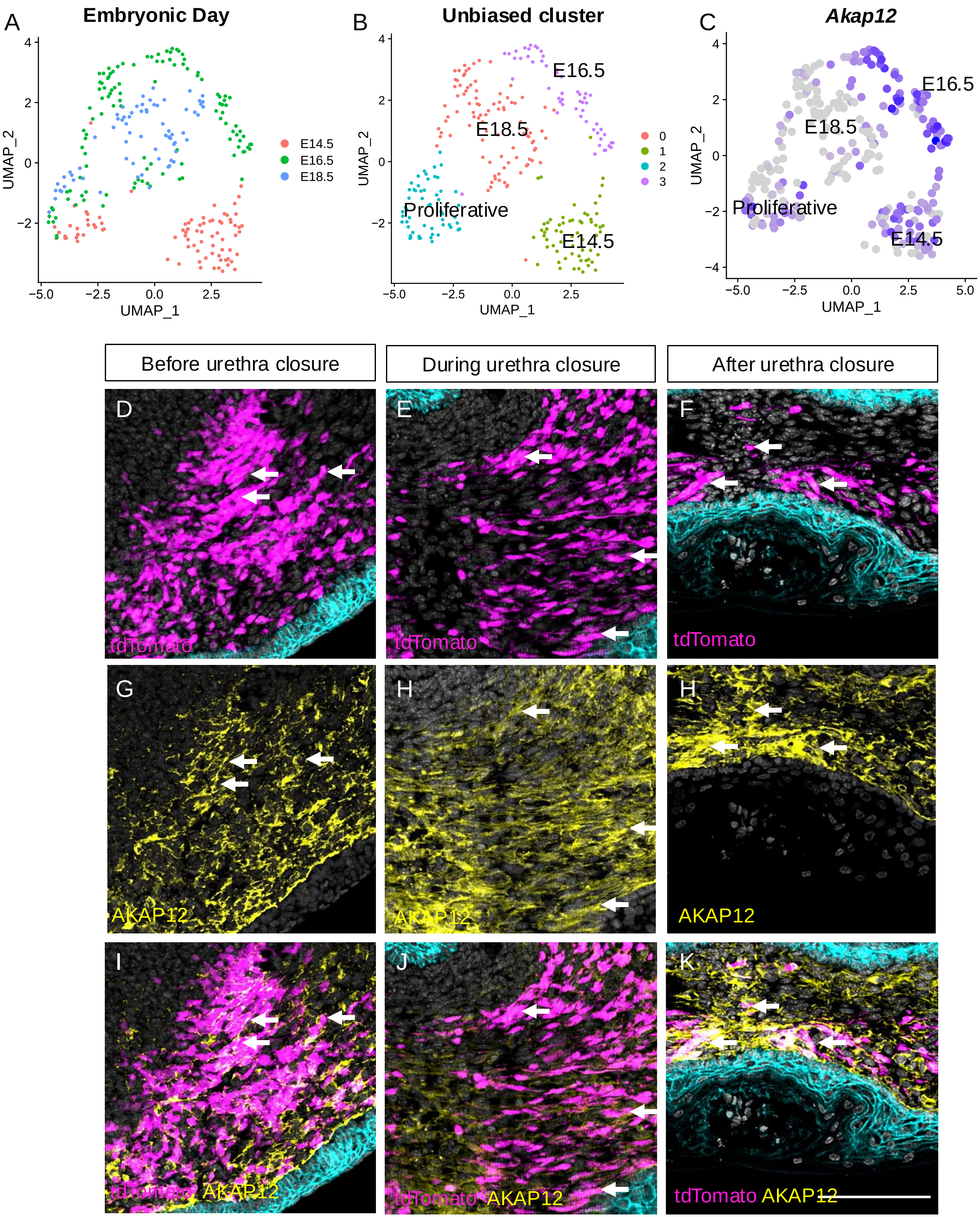

A
Embryonic Day
E14.5
E16.5
E18.5
UMAP_1
UMAP_2
B
Unbiased cluster
E16.5
E18.5
Proliferative
E14.5
0
1
2
3
C
Akap12
Before urethra closure
During urethra closure
After urethra closure
D
E
F
tdTomato
G
H
AKAP12
I
J
K
tdTomato AKAP12

Figure S2. Investigation of *Nr5a1*$^{tdTomato+}$heterogenitity. (A) UMAP of *Nr5a1*$^{tdTomato+}$ cells with points colored by the embryonic day. (B) UMAP of *Nr5a1*$^{tdTomato+}$ cells with points colored by unbiased clustering from Seurat. (C) UMAP of *Akap12* expression within the *Nr5a1*$^{tdTomato+}$ cells. Grey= little to no expression and blue = high expression. (D-F) High magnification of tdTomato localization within the penis at E14.5, E16.5, and E18.5. (G-I) High magnification of AKAP12 expression within the genitalia. White arrows indicated the same cells from the tdTomato staining. (I-J) tdTomato and AKAP12 expression within the penis at E14.5, E16.5, and E18.5.

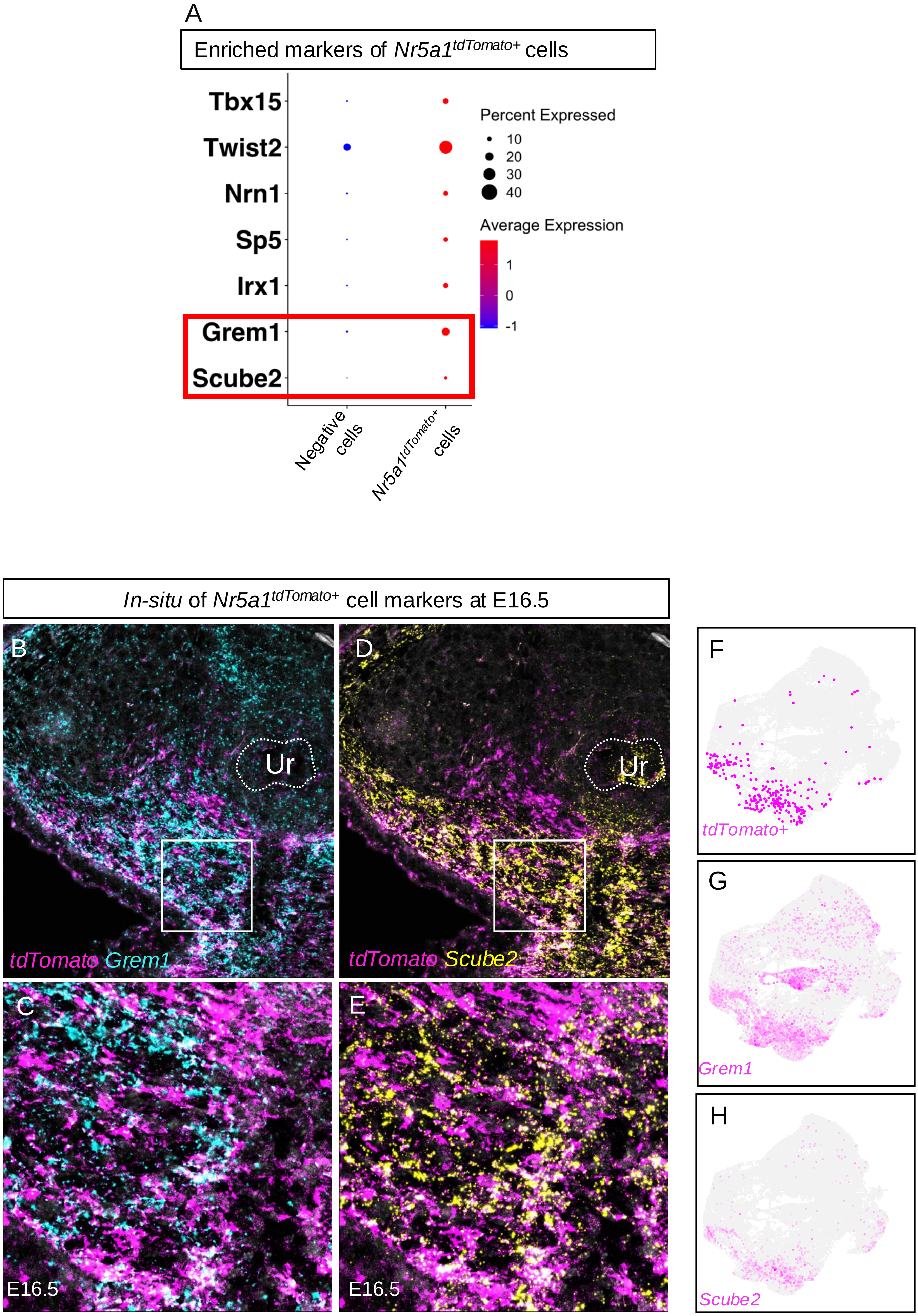
A
Enriched markers of Nr5a1tdTomato+ cells
Tbx15
Twist2
Nrn1
Sp5
Irx1
Grem1
Scube2
Percent Expressed
10
20
30
40
Average Expression
1
0
-1
Negative cells
Nr5a1tdTomato+ cells
In-situ of Nr5a1tdTomato+ cell markers at E16.5
B
Ur
tdTomato Grem1
D
Ur
tdTomato Scube2
C
E16.5
E
E16.5
F
tdTomato+
G
Grem1
H
Scube2

Figure S3. Other markers *Nr5a1$^{tdTomato+}$* cell population. (A) Dotplot of significant *Nr5a1$^{tdTomato+}$* cell markers with the size of the dot representing the percentage of cells expressing the gene and the color of dot showing relative expression (red=high and blue = low). (B-E) Immunofluorescence of *Nr5a1$^{tdTomato+}$* cells (magenta) , *Grem1* (cyan **Panels B and C**) and *Scube2* (yellow **Panels D and E**) in penis sections at E16.5. (F-H) UMAP of *tdTomato*, *Scube2* gene expression in E16.5 penis. Ur = urethra, Scale bars= 50µm

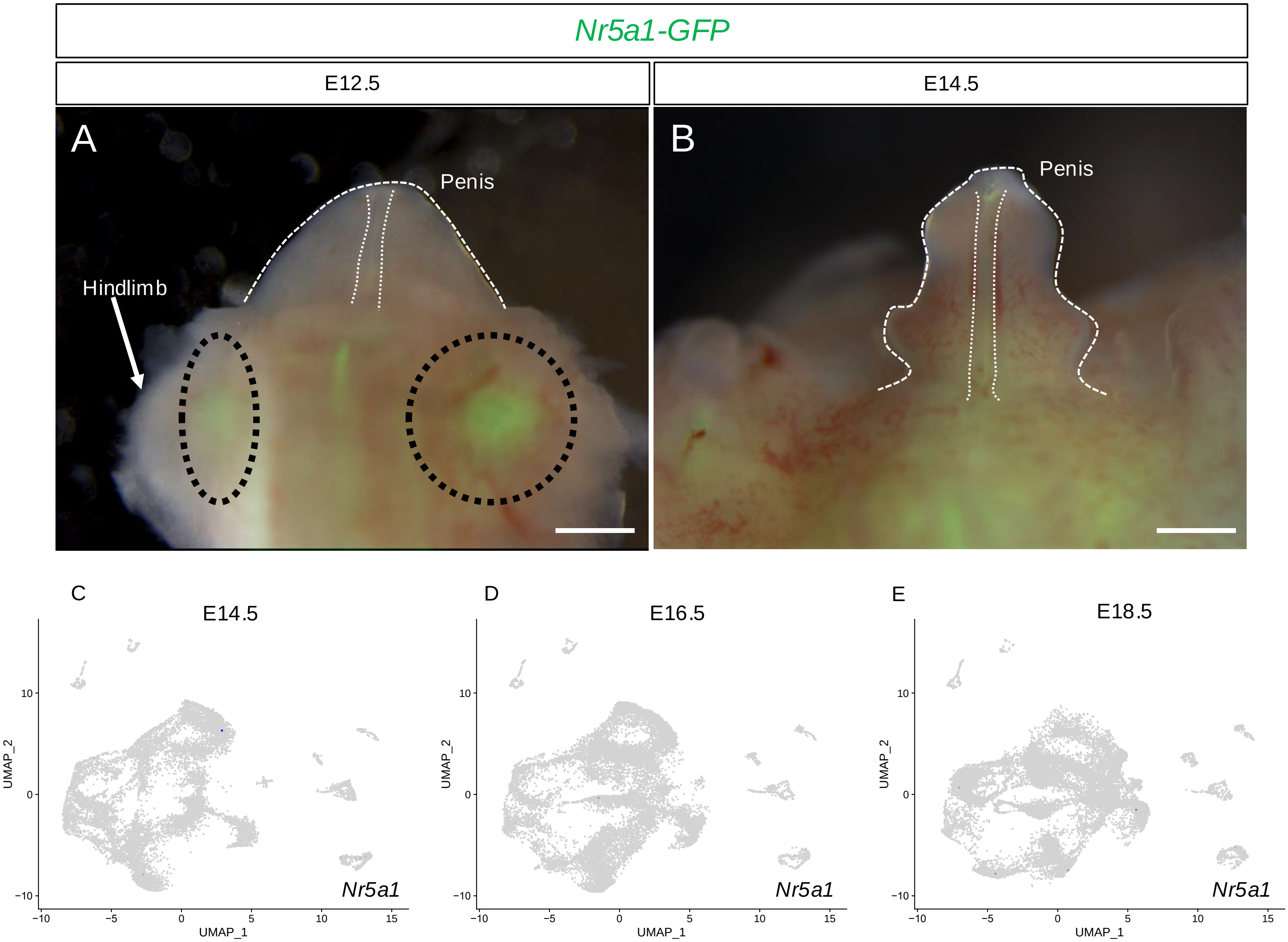

Nr5a1-GFP
E12.5
E14.5
A
Penis
Hindlimb
B
Penis
C
E14.5
D
E16.5
E
E18.5
UMAP_2
UMAP_1
Nr5a1
Nr5a1
Nr5a1

Figure S4. Investigating active expression of *Nr5a1* in penis development. (A and B) Lower half of *Nr5a1-GFP* mouse embryo at E12.5 and E14.5. White solid line outlines the genitalia. Black dotted circles outline the active expressing regions of GFP in the E12.5 embryo. (C-E) Single cell mRNA sequencing gene expression UMAPs for *Nr5a1* with grey dots displaying no expression and dark blue showing high expression. Scale bars = 800µm

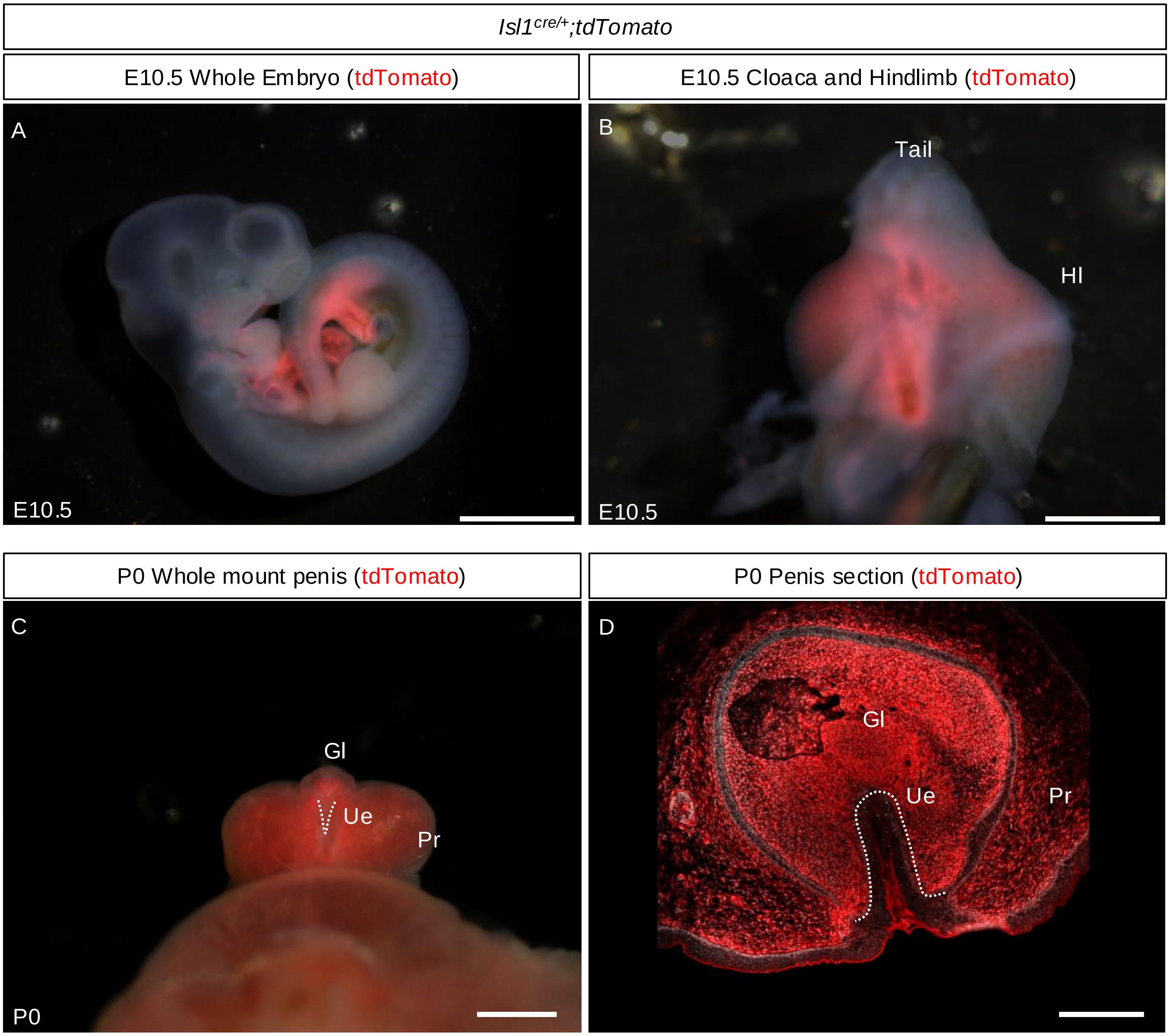

Isl1$^{cre/+}$;*tdTomato*
E10.5 Whole Embryo (tdTomato)
E10.5 Cloaca and Hindlimb (tdTomato)
A
E10.5
B
Tail
Hl
E10.5
P0 Whole mount penis (tdTomato)
P0 Penis section (tdTomato)
C
Gl
Ue
Pr
P0
D
Gl
Ue
Pr

Figure S5. Validation that *Isl1Cre* targets the external genitalia prior to genitalia formation. (**A** and **B**) Endogenous fluorescence of tdTomato at E10.5. **(C)** Whole mount image of external genitalia at P0 with endogenous red fluorescence. (**D**) histological section of P0 genitalia with red representing endogenous fluorescence. Hl=Hindlimb, Gl = Glans, Ue = urethra, and Pr= Prepuce. Scale bar for A = 1.4 mm, B = 540 µm, C = 800 µm, and D = 100µm

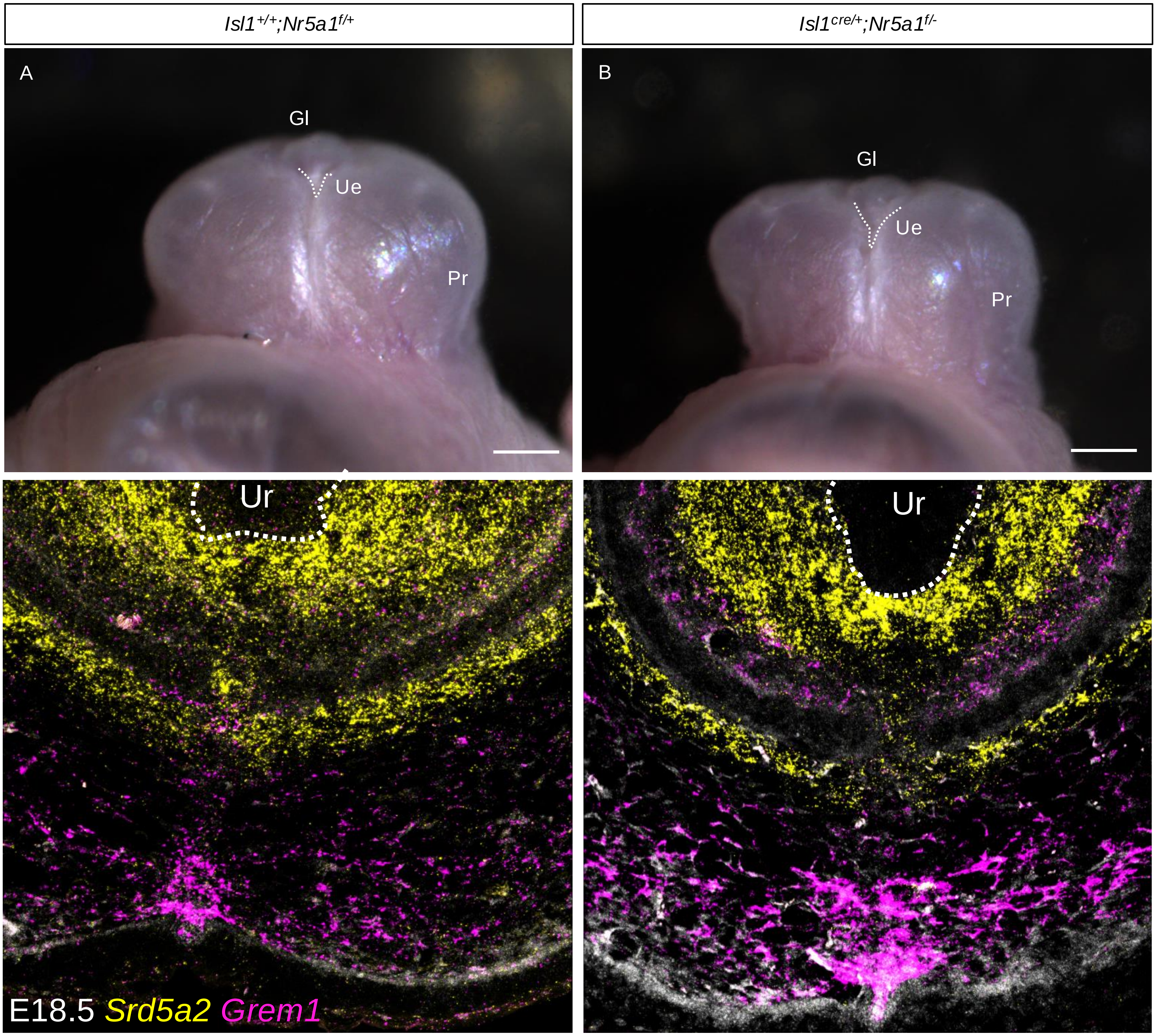

Isl1+/+;Nr5a1f/+
Isl1cre/+;Nr5a1f/-
A
Gl
Ue
Pr
B
Gl
Ue
Pr
Ur
Ur
E18.5 Srd5a2 Grem1

Figure S6. Knockout of *Nr5a1*, the gene, in the penis. (A and B) Whole mount images of control (A) and knockout (B) mouse penises. White dotted line represents the open urethra. (**C** and **D**) RNAscope for *Srd5a2* (yellow) and the *Nr5a1*$^{tdTomato+}$ marker, *Grem1* *(*magenta*)* on penis sections with the dotted line representing the urethra epithelium. Ur= urethra,. Whole mount scale bars = 200 µm and microscope scale bars = 100 µm

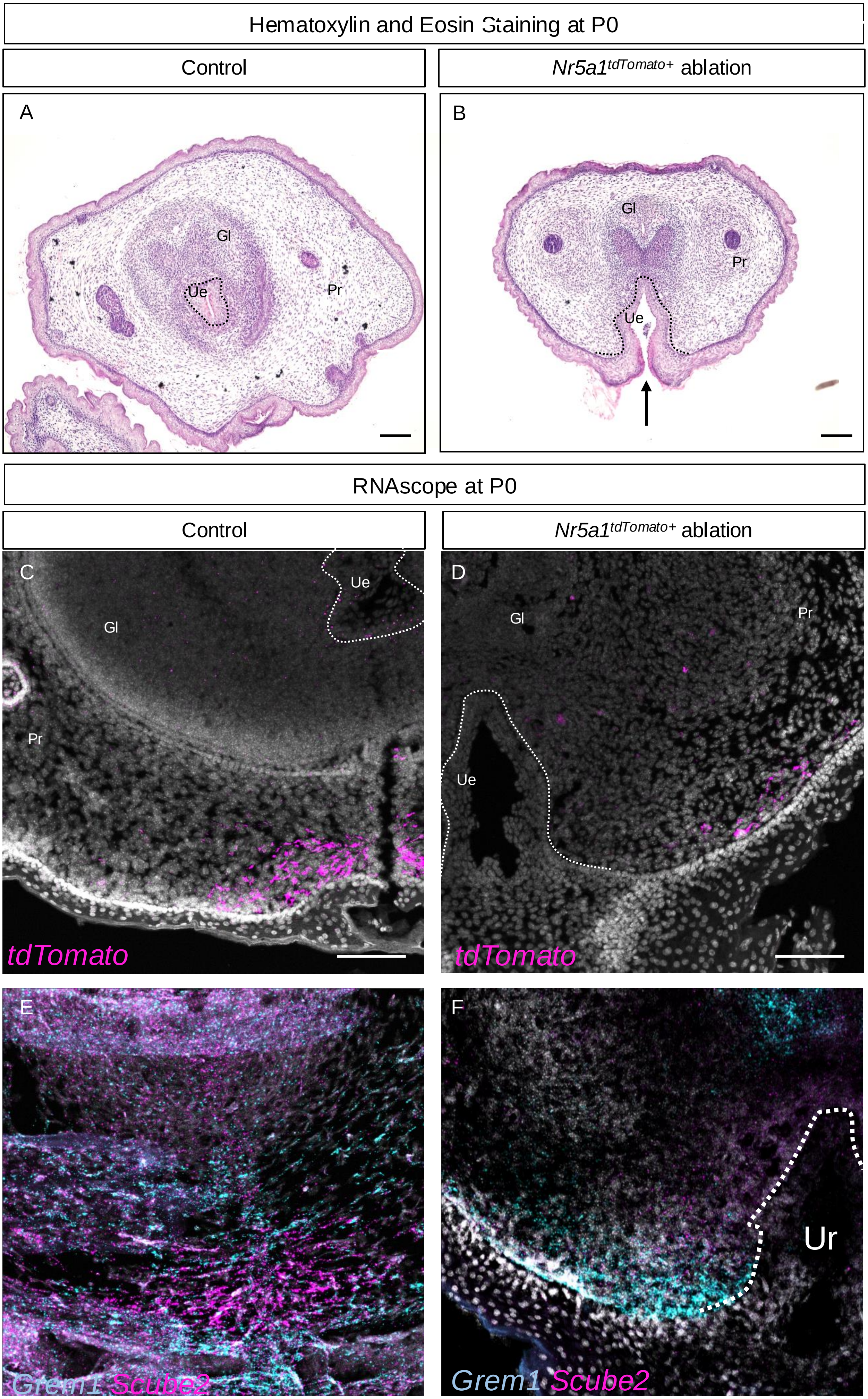

Hematoxylin and Eosin Staining at P0
Control
Nr5a1tdTomato+ ablation
A
Gl
Ue
Pr
B
Gl
Pr
Ue
RNAscope at P0
Control
Nr5a1tdTomato+ ablation
C
Ue
Gl
Pr
tdTomato
D
Gl
Pr
Ue
tdTomato
E
Grem1 Scube2
F
Ur
Grem1 Scube2

Figure S7. Validation of *Nr5a1*$^{tdtomato+}$ in the penis. (A and B) Hematoxylin and Eosin staining of penis sections from control and *Nr5a1*$^{tdTomato+}$ cell ablated embryos with the black dotted lines indicating the urethra and the black arrow indicating the open urethra. **(C** and **D**) RNAscope for *tdTomato* in control and *Nr5a1*$^{tdTomato+}$ cell ablated penis sections. (**E** and **F**) RNAscope for *Nr5a1*$^{tdTomato+}$ cells, *Grem1* (cyan) and *Scube2* (magenta). Gl = Glans, Pr= Prepuce, and Ue = Urethra.

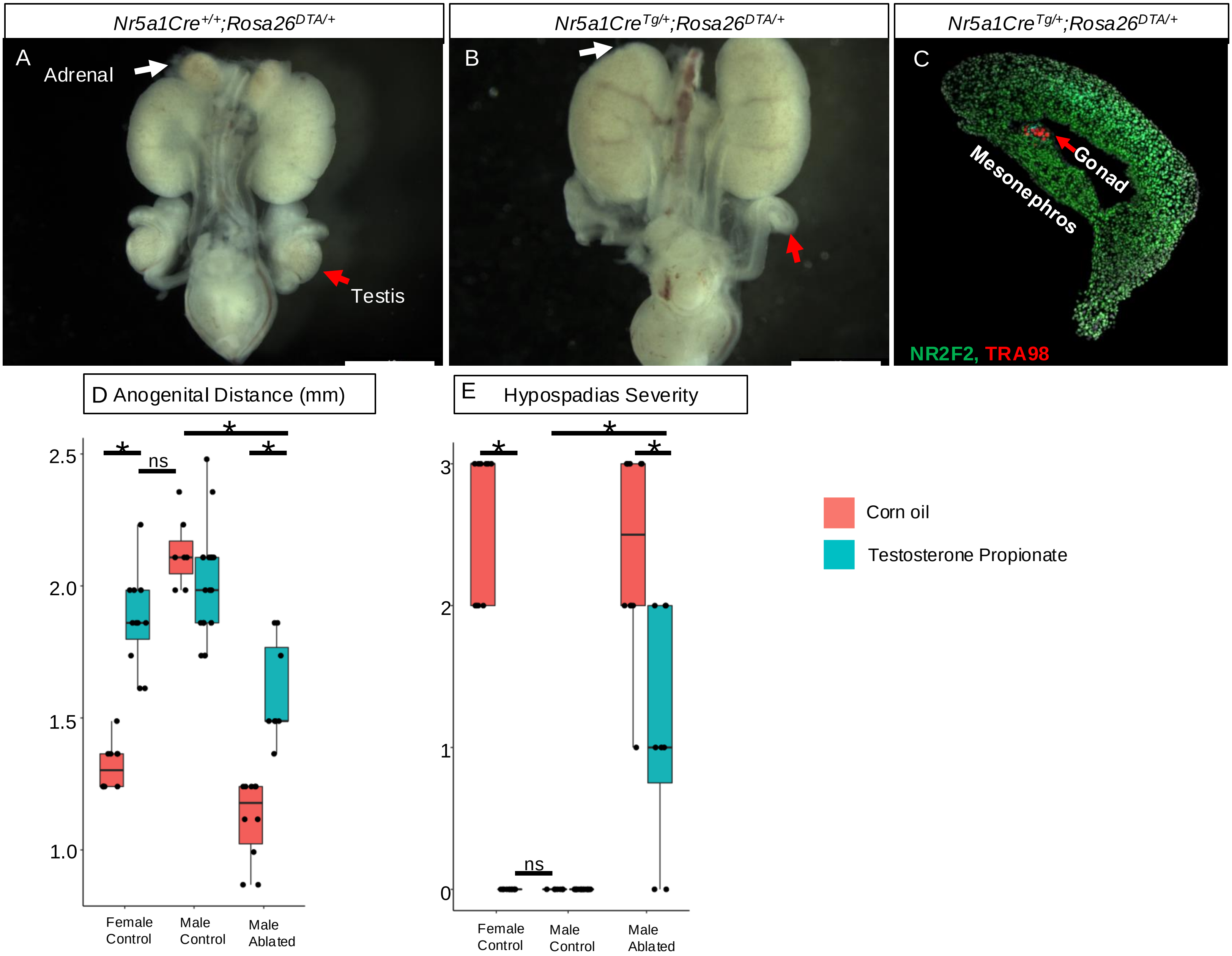

Nr5a1Cre+/+;Rosa26DTA/+
Nr5a1CreTg/+;Rosa26DTA/+
Nr5a1CreTg/+;Rosa26DTA/+
A
Adrenal
Testis
B
C
Gonad
Mesonephros
NR2F2, TRA98
D Anogenital Distance (mm)
2.5
2.0
1.5
1.0
ns
Female Control
Male Control
Male Ablated
E Hypospadias Severity
3
2
1
0
ns
Female Control
Male Control
Male Ablated
Corn oil
Testosterone Propionate

Figure S8. Investigation of *Nr5a1* cell ablation in other organs. (A and B) Whole mount image of the urogenital complex of control and ***Nr5a1* cell** ablated mice (exact name in figure). White arrow indicates positioning of the adrenal and red arrow indicated positioning of the testis. (**C)** Immunofluorescent image of a $Nr5a1^{tdtomato+}$ cell ablated gonad with red labeling TRA98+ germ cells and green labeling NR2F2+ interstitial cells of the mesonephros (and a few from the gonad I suppose). The red arrow designates the gonad. (**D**) Anogenital distance length for control and $Nr5a1^{tdtomato+}$ cell ablated mice exposed to either corn oil (red) or testosterone propionate (teal). Asterisk indicate $p<0.05$. (**E**) Hypospadias severity scoring for control and $Nr5a1^{tdtomato+}$ cell ablated mice exposed to either corn oil (red) or testosterone propionate (teal). A score of 3 represents severe hypospadias and a score of 0 represents no hypospadias. Asterisk indicate $p<0.05$. Whole mount scale bar = 2mm

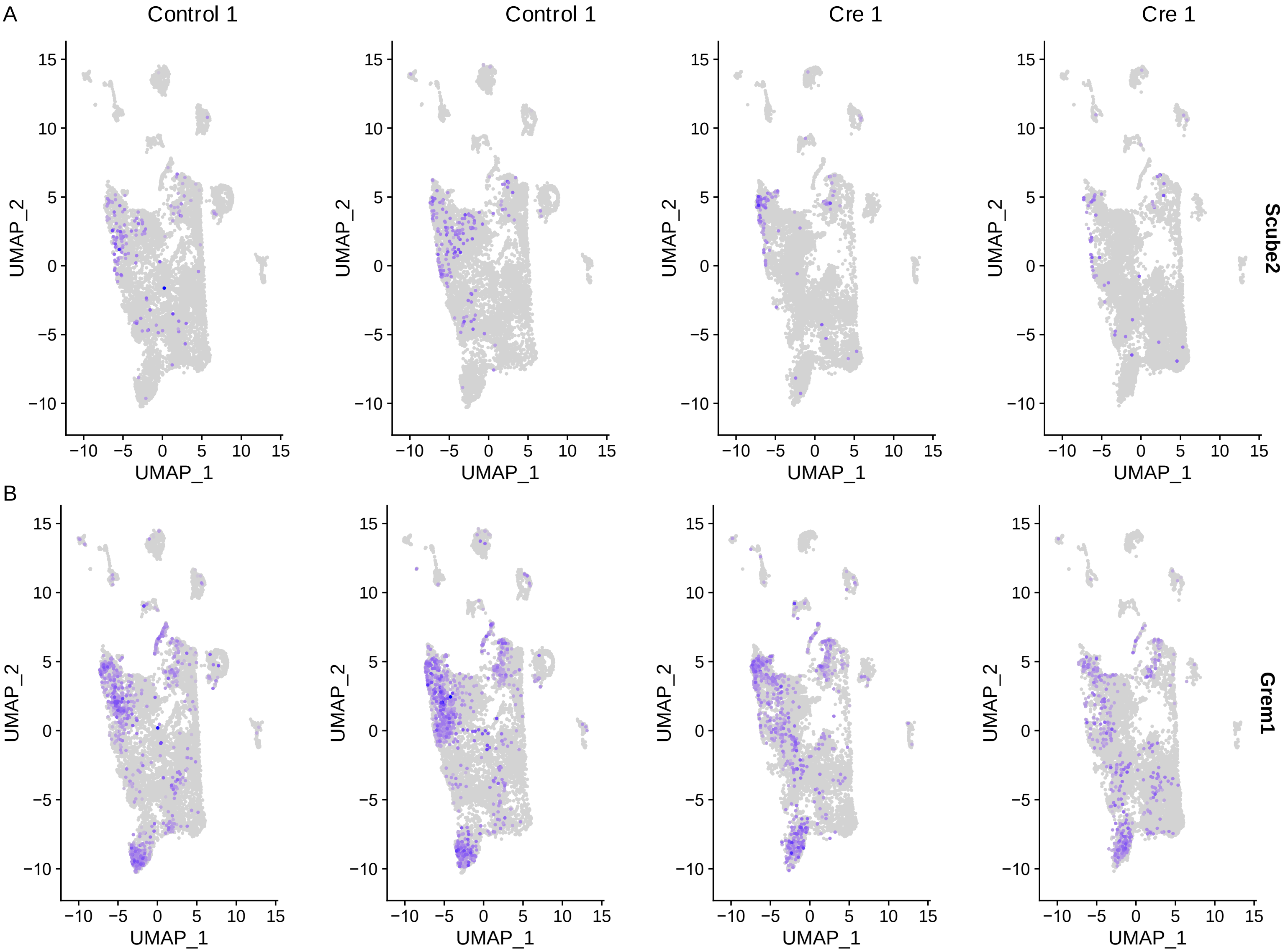

A
Control 1
Control 1
Cre 1
Cre 1
Scube2
B
Grem1
UMAP_1
UMAP_2

Figure S9. Expression of *Nr5a1*$^{tdTomato+}$ enriched genes. (**A**) Expression of *Scube2* in control and Cre+ (*Nr5a1*$^{tdTomato+}$ cell ablated) mice. (**B**) Expression of *Grem1* in control and Cre+ mice.

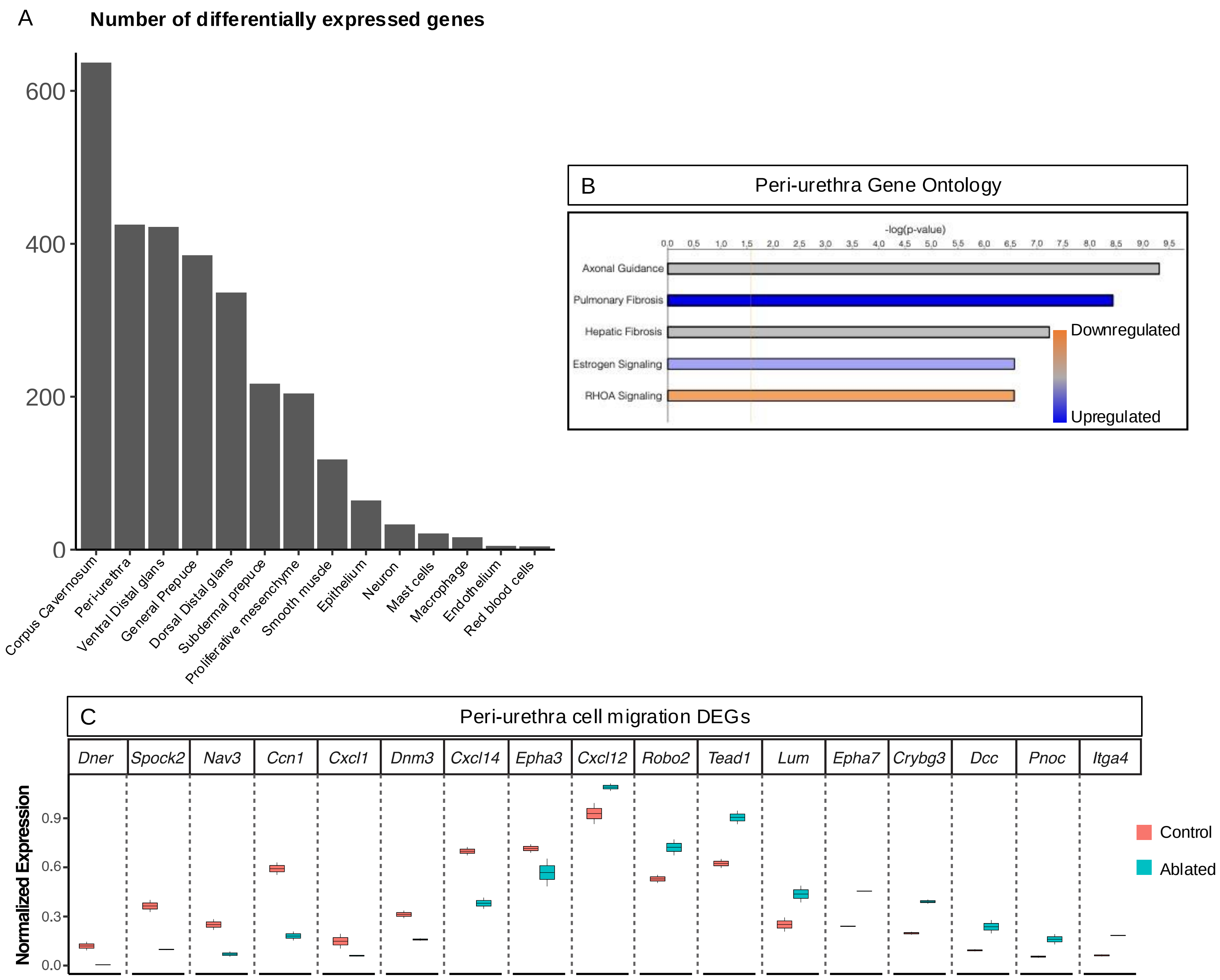
A
Number of differentially expressed genes
600
400
200
0
Corpus Cavernosum
Peri-urethra
Ventral Distal glans
General Prepuce
Dorsal Distal glans
Subdermal prepuce
Proliferative mesenchyme
Smooth muscle
Epithelium
Neuron
Mast cells
Macrophage
Endothelium
Red blood cells
B
Peri-urethra Gene Ontology
-log(p-value)
0.0 0.5 1.0 1.5 2.0 2.5 3.0 3.5 4.0 4.5 5.0 5.5 6.0 6.5 7.0 7.5 8.0 8.5 9.0 9.5
Axonal Guidance
Pulmonary Fibrosis
Hepatic Fibrosis
Estrogen Signaling
RHOA Signaling
Downregulated
Upregulated
C
Peri-urethra cell migration DEGs
Dner
Spock2
Nav3
Ccn1
Cxcl1
Dnm3
Cxcl14
Epha3
Cxcl12
Robo2
Tead1
Lum
Epha7
Crybg3
Dcc
Pnoc
Itga4
Normalized Expression
0.9
0.6
0.3
0.0
Control
Ablated

Figure S10. Impacts of *Nr5a1* cell ablation on other penis cell populations. (**A**) Differential gene expression between wildtype and *Nr5a1*$^{tdtomato+}$ cell ablated mice for each penis cell population. (**B and C**) Gene ontology of the differential expressed genes found in the peri-urethra and prepuce. Grey bars represent no discernable up or down regulation of the pathway, increasingly red bars indicate elevation of the pathway. (**D** and **E**) Gene expression boxplots of control (red) and ablated (teal) for significantly altered genes in the prepuce (D) and peri-urethra (E).

Table S1. Sequencing results from *Nr5a1*$^{tdtomato+}$ cell ablated single cell

Sample	Raw.Reads	Barcode	Mapped	Saturation	Cells	Reads/ Cell	Median umi/cell	Median genes/cell
DTA 1	163,797,148	93%	87%	27%	11,511	14,230	3,578	1,478
DTA 2	159,515,741	96%	89%	28%	11,349	14,055	3,661	1,592
MALE 1	221,873,673	97%	86%	35%	12,570	17,651	4,940	1,816
MALE 2	153,731,758	96%	87%	25%	11,613	13,238	3,992	1,627

Table S2. Antibodies

**Target**	**Company**	**Catalog number**	**Concentration**
AKAP12	Collaborator	N/A	1:300
FOXL2	Abcam	Ab5096	1:300
EGFR	EGFR	ab32077	1:400
FOXA1	FOXA1	Ab170933	1:200
dsRED	Clontech	632496	1:1000
E-cadherin	Abcam	Ab11512	1:500
Alexafluor 488	Invitrogen	A21208/A21206	1:400
Alexfluor 568	Invitrogen	A10042	1:400
Alexfluor 647	Life Technology	A21084	1:400
